# Exhaled breath analysis for gastric cancer diagnosis in Colombian patients

**DOI:** 10.18632/oncotarget.25331

**Published:** 2018-06-22

**Authors:** Cristhian Manuel Durán-Acevedo, Aylen Lisset Jaimes-Mogollón, Oscar Eduardo Gualdrón-Guerrero, Tesfalem Geremariam Welearegay, Julián Davíd Martinez-Marín, Juan Martín Caceres-Tarazona, Zayda Constanza Sánchez-Acevedo, Kelvin de Jesus Beleño-Saenz, Umut Cindemir, Lars Österlund, Radu Ionescu

**Affiliations:** ^1^ Multisensor System and Pattern Recognition Research Group (GISM), Electronic Engineering Program, Universidad de Pamplona, Pamplona, Colombia; ^2^ Department of Electronics, Electrical and Automatic Engineering, Rovira i Virgili University, Tarragona, Spain; ^3^ GASTROSUR S.A., Universidad Nacional de Colombia, Facultad de Medicina, Bogotá, Colombia; ^4^ Hospital Universitario la Samaritana, Bogotá, Colombia; ^5^ Mechatronics Engineering Department, Universidad Autónoma del Caribe, Barranquilla, Colombia; ^6^ Molecular Fingerprint Sweden AB, Uppsala, Sweden; ^7^ Department of Solid State Physics, The Ångström Laboratory, Uppsala University, Uppsala, Sweden

**Keywords:** gastric cancer, breath analysis, volatile organic compounds, biomarkers, chemical gas sensor

## Abstract

We present here the first study that directly correlates gastric cancer (GC) with specific biomarkers in the exhaled breath composition on a South American population, which registers one of the highest global incidence rates of gastric affections. Moreover, we demonstrate a novel solid state sensor that predicts correct GC diagnosis with 97% accuracy. Alveolar breath samples of 30 volunteers (patients diagnosed with gastric cancer and a controls group formed of patients diagnosed with other gastric diseases) were collected and analyzed by gas-chromatography/mass-spectrometry (GC-MS) and with an innovative chemical gas sensor based on gold nanoparticles (AuNP) functionalized with octadecylamine ligands. Our GC-MS analyses identified 6 volatile organic compounds that showed statistically significant differences between the cancer patients and the controls group. These compounds were different from those identified in previous studied performed on other populations with high incidence rates of this malady, such as China (representative for Eastern Asia region) and Latvia (representative for Baltic States), attributable to lifestyle, alimentation and genetics differences. A classification model based on principal component analysis of our sensor data responses to the breath samples yielded 97% accuracy, 100% sensitivity and 93% specificity. Our results suggest a new and non-intrusive methodology for early diagnosis of gastric cancer that may be deployed in regions lacking well-developed health care systems as a prediagnosis test for selecting the patients that should undergo deeper investigations (*e.g.*, endoscopy and biopsy).

## INTRODUCTION

Gastric cancer (GC) is one of the most lethal cancers worldwide [1, 2]. The highest incidence regions are Eastern Asia, Baltic States and Latin America [3–5]. In the particular case of Colombia, gastric cancer is the primary cause of death from malign tumors in both genders [6]. The most important factor that determines such high number of deaths from GC is associated with late diagnosis, due to its asymptomatic incipiency, which avoids the prescription of early treatments. Today, endoscopy and biopsy are the gold standard tests used for detecting and diagnosing GC, but these procedures are time-consuming, unpleasant and not completely without risks for the patients. Further, they are expensive and therefore unaffordable for mass screening in developing countries where GC prevails, and they are not able to provide an early diagnosis of the disease. For this reason, alternative diagnosis methods are very much needed. In this regard, exhaled breath analysis has gained large scientific and clinical interest for medical diagnosis in recent years [7]. Molecules in the exhaled breath are produced by malignant cells of the tumor and are transported in the blood to the lungs, where they are exhaled by the bronchia alveolus. Pioneer studies performed on study populations from Latvia and China [8, 9] demonstrated the utility of breath analysis for the diagnosis of gastric cancer, although they also revealed the existence of geographical variations in the breath volatiles pattern associated with GC, mainly due to genetics and nutrition factors [8].

In the present study, we report for the first time the identification of a breath volatiles pattern associated with GC in a South American population, and demonstrate new chip-based sensor technologies amenable for early screening purposes that correctly predicted GC with 97% accuracy. We compare our results with those obtained in the previous studies performed on Chinese and Latvian populations.

## RESULTS

### Breath biomarkers of gastric cancer identified in Colombian patients

The GC-MS analysis of the breath samples identified six compounds whose concentrations were statistically different for patients diagnosed with GC compared with controls group of patients diagnosed with other gastric diseases (see the full volunteers list in Table 2 from Materials and Methods section).

These compounds, shown in Table 1, represent putative breath biomarkers of GC for Colombian patients.

**Table 1:** Breath biomarkers for Colombian patients diagnosed with gastric cancer

Group		Compound	CAS N°	Structural formula	P-value
**GASTRIC CANCER PATIENTS**	VOC1	Trans-2, 2-dimethyl-3-decene	**55499-02-0**	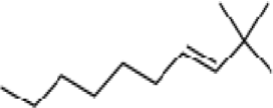	0.006
	VOC2	Octadecane	**593-45-3**	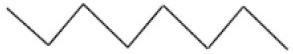	0.022
	VOC3	M-xylene	**108-38-3**	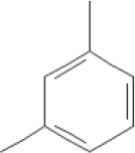	0.029
	VOC4	Hexadecane	**544-76-3**	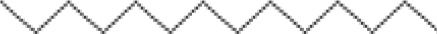	0.031
**CONTROL PATIENTS**	VOC5	1-Cyclohexyl-2-(cyclohexylmethyl) pentane	**55030-21-2**	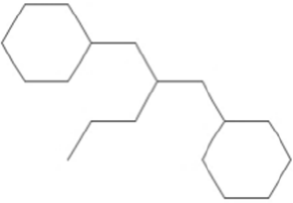	0.034
	VOC6	Eicosane	**112-95-8**	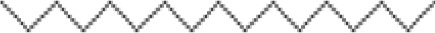	0.045

**Figure 1:**
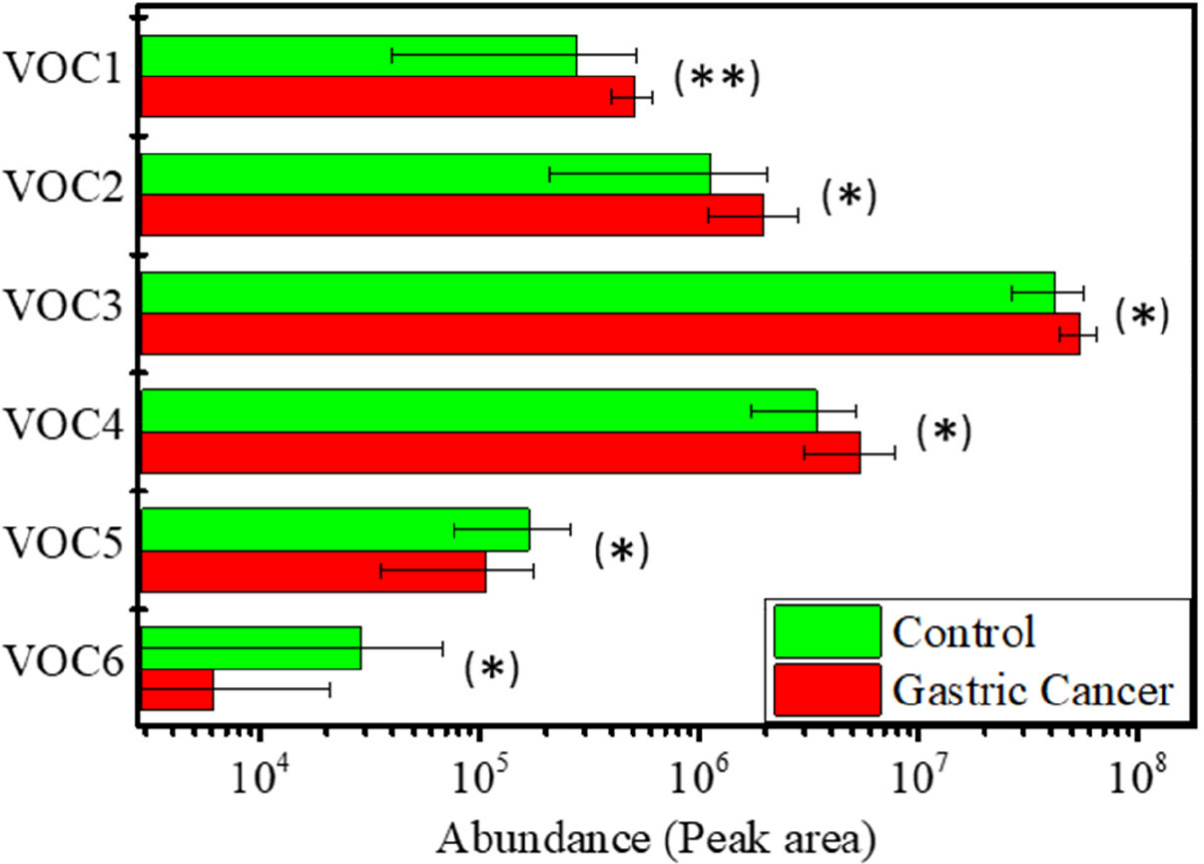
Biomarkers abundances in the breath of the GC and Control patients Error bars represent the standard error of the mean. (^*^) Statistically significant difference (p < 0.05) between GC and Control groups; (^**^) statistically significant difference (p < 0.01) between GC and Control groups.

Four of these biomarkers were found in increased concentrations in the breath of the GC patients, while the concentrations of the other two biomarkers decreased in the case of the same patients (Figure 1). A detailed analysis of the abundances of each biomarker in the breath sample of all volunteers included in this study is presented in the Supplemental Material (Supplementary Figures 1-6).

**Figure 2:**
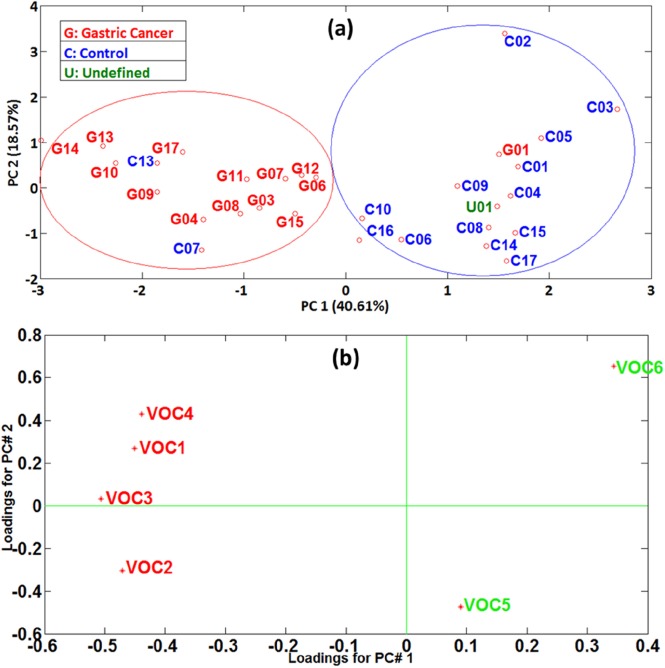
**(a)** PCA scores plot performed with biomarkers abundances. GC: red labels; Control: blue labels; Undefined: green label; **(b)** PCA loadings plot performed with biomarkers abundances. Biomarkers with increased concentration in GC patients' breath: red numbers; Biomarkers with increased concentration in Controls' breath: green numbers.

Figure 2 shows the result of the PCA analysis performed with the values of the abundances of these biomarkers in the volunteers breath. A good classification was obtained between the GC and control groups, with only two control volunteers (C07 and C13) and one gastric cancer patient (G01) misclassified, Figure 2(a), providing 90% classification accuracy, 93% sensitivity and 87% specificity. The loadings plot presented in Figure 2(b) confirmed that the contribution of all biomarkers is important for the results obtained. Moreover, a very clear difference could be observed on PC1 loadings between the biomarkers whose concentration increased or decreased in the breath of the GC patients, which were plotted in the negative or positive region of the axis, respectively. The variance captured by each PC is presented in Supplementary Table 1 from the Supplemental Material.

### Chemical gas sensor results

**Figure 3:**
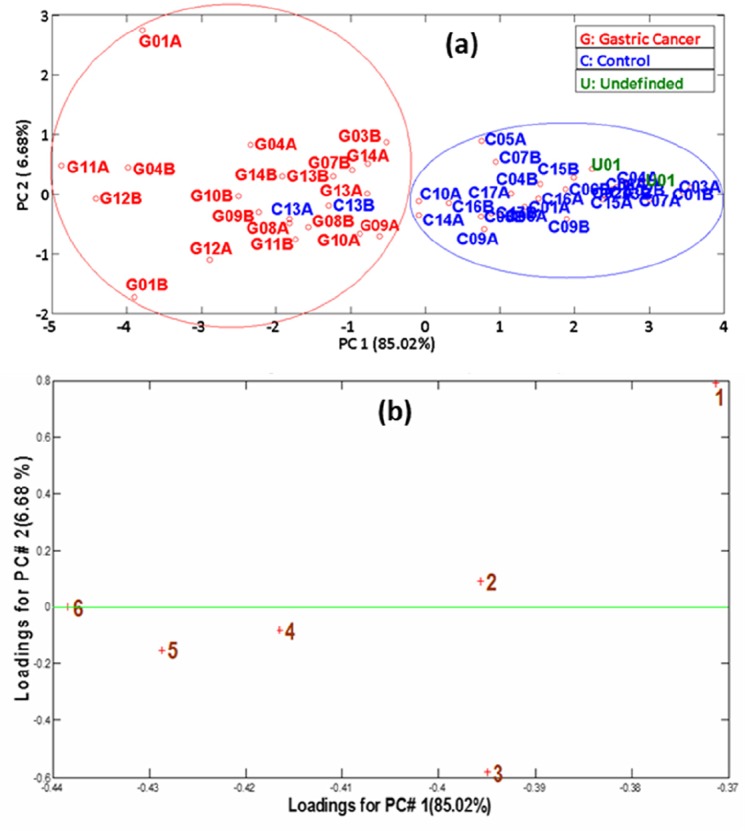
**(a)** PCA scores plot obtained from sensor's responses. GC: red labels; Controls: blue labels; Undefined: green label. Labels A and B after the patient number correspond to two different samples provided by the same patient; **(b)** PCA loadings plot obtained from sensor's responses. The numbers in the graph represent the different sensor's features.

Figure 3 shows the result of the PCA analysis performed using the six features extracted from the responses of the solid state AuNP-octadecylamine ligand chemical gas sensor (*see* Materials and Methods section) to two duplicate breath samples provided by each patient from the study. Figure 3(a) illustrates an excellent classification of all GC patients, while the two samples corresponding to the same control patient (C13) were misclassified as GC, which overall yield 97% accuracy of samples classification, 100% sensitivity and 93% specificity. The loadings plot presented in Figure 3(b) confirmed that the contribution of all six sensor's features employed to build the PCA model are important and they do not overlap, not multiplying thus the same information.

## DISCUSSION

### GC biomarkers

Gastric cancer is associated with several risk factors, among which the most important, as we know today, are the excessive consumption of salt and smoked foods, smoking habit, abuse of alcohol, infection with *Helicobacter pylori*, environmental conditions, and working environment [10, 11]. Genetic factors are not a determining factor for developing GC, which explains the low percentage of patients from our study with first-degree relatives suffering from GC. Nevertheless, we should note that the possibility of being diagnosed with GC drastically increases after the age of 50 (although late diagnosis could play an important role here), and GC is also known to be more common for men than women. Therefore, patients age and gender and the infection with *Helicobacter pylori* were considered as confounding factors in this study, as well as the smoking habit that has an important influence on exhaled breath composition.

Regarding the provenance of the patients in this study, many of them live in the countryside and predominantly use wood for cooking, which usually exposes them to soot and cocktails of products formed during incomplete, low-temperature combustion, such as carbon monoxide, benzene, butadiene, formaldehyde, together with a high concentration of particles that contain a variety of alkanes, alkenes and polycyclic aromatic hydrocarbons, all recognized as being harmful for human health. Among these toxic substances alkanes with hydrocarbonated chains from C15 to C35 are typically found, which could explain the presence of the biomarkers *hexadecane* and *octadecane* in the GC group, while *eicosane* and *1-cyclohexyl-2-(cyclohexylmethyl) pentane* were identified as biomarkers in the control group (*i.e.*, patients with other gastric diseases) [12].

Other relevant factor for GC is the occupational exposure, being the excavators and pavers, forestry workers, electric and electronic workers, motor transport workers, and food industry employees the categories with elevated risks. The substances most plausibly associated with gastric cancer are crystalline silica, leaded gasoline, grain dust, lead dust, zinc dust, hydraulic fluids, and glycol ethers [13]. The frequency, duration and intensity of the exposures to these substances in the working environment, as well as the way they enter into the organism (inhalation, ingestion or dermic absorption), plays a role for initiation of this disease. In Colombia this risk is highly increased by the lack of appropriate and sufficient protection equipment employed by the workers during their normal activity [14].

Farmers that are exposed to harmful substances present in chemical products (*e.g.* fertilizers, insecticides, fumigants and herbicides) can be also specifically affected. One component in the pesticides is *m*-xylene, which is also widely used as solvent, cleaning agent, paintings dissolvent, in the chemical industry and in the manufacturing industry of plastic products, synthetic fibers, rubber and leather products. Building workers are exposed to m-xylene, as they have contact with solvents and thinners for different kind of paintings (spray lacquer, varnish) which all contain m-xylene. Chronic exposure to xylenes can cause depression, anemia, hemorrhage of the mucous membranes, hyperplasia of the bone marrow, increase of the size of the liver and nephrosis [15]. However, according to experimental studies on animals, there is no enough evidence to classify xylenes (meta, ortho and para) as carcinogen agents, and they are classified in the Group 3 (not classifiable as carcinogenicity to humans) by the International Agency for the Research of Cancer (IARC) and the Environment Protection Agency (EPA) [16].

Environmental air pollutants comprise volatile organic compounds (VOCs) with photochemical ozone creation potential (POCP), which produce ozone at the ground level known as “summer smog” or photochemical smog due to their ability to form ozone in relation to ethylene, which happens when they are mixed with nitrogen oxides. Among these substances, the alkenes have the highest POCP. One of the identified biomarkers associated with GC, *trans-2, 2-dimethyl-3-decene*, belongs to this group of compounds [17].

The pathophysiology to explain the GC related biomarkers identified in this study and their biochemical mechanism of production is not yet well understood. However, research studies suggested that cancer development may involve increased oxidative stress and upregulation of cytochrome p-450 enzymes under metabolic stress due to carcinogenesis [18–20]. As the human organism relies on mitochondrial oxidative phosphorylation for energy production, a leaked oxygen radical leads to the formation of highly reactive oxygen species such as superoxide, hydrogen peroxide and hydroxyl radical, which are highly toxic for cellular constituents. The reactive radicals tend to provoke lipid peroxidation and convert the polyunsaturated fatty acids into various volatile alkanes that are released in the breath shortly after their production. Straight chain aliphatic alkanes such as *octadecane* (C8 carbon chain length), *hexadecane* (C16 carbon chain length) and *eicosane* (C20 carbon chain length) are generated from lipid peroxidation [21–23]. While octadecane and hexadecane were found elevated in the breath of GC patients, the concentration of eicosane decreased; this could be related to the fact that the long chain of this aliphatic alkane might be metabolised by the cytochrome P450 into smaller molecules such as alcohols, and therefore its concentration is reduced in the exhaled breath of GC patients [20–23]. *Trans-2, 2-dimethyl-3-decene* might be generated as an intermediate product of conjugated dienes once the chain reaction of lipid peroxidarion has been initiated [20]. Finally, the metabolic origin of *1-cyclohexyl-2-(cyclohexylmethyl) pentane* is difficult to elucidate, while *m*-xylene is an aromatic hydrocarbon of exogenous origin.

The three patients misclassified by the PCA model based on the GC breath biomarkers identified in this study belong to the control patients C07 (the only control patient diagnosed with ulcer) and C13 (who performed double exhalation because of physical weakness) and to the gastric cancer patient G01 (with very critical health condition). The undefined patient U01 (GC patient that responded well to chemotherapy) was plotted in the Controls group. Corroborating the results obtained in the present study with those obtained on study populations from China and Latvia [8, 9], it is evident that geographic factors associated with lifestyle, alimentation and genetics (gastrointestinal flora) have an important effect on the metabolomics pathway of the gastric cancer disease. The GC patients from the Chinese and Latvian studies had elevated levels of a wide variety of organic compounds, including ketones (6-methyl-5-hepten-2-one, both China and Latvia), aldehydes (nonanal, Latvia), alcohols (2-butoxy-ethanol, China, and 2-ethyl-1-hexanol, Latvia), alkenes (isoprene, China), nitriles (2-propenenitrile, China), and aromatic compounds (furfurali China, and styrene, Latvia). In the present study we could also identify compounds from the alkenes (trans-2, 2-dimethyl-3-decene) and aromatic compounds (*m*-xylene) groups, but also compounds form the chemical group of alkanes (hexadecane and octadecane), which were not identified in the Chinese or Latvian populations. Nevertheless, the results of this study performed on patients from Colombia revealed different GC biomarkers as compared with the studies performed on patients from Eastern Asia and the Baltic States.

### GC diagnosis with the chemical gas sensor

A high diagnosis accuracy of GC (97%) was obtained with the chemical gas sensor employed in this study, which is on par with the accuracy of the endoscopy test that represents the current standard diagnosis procedure for GC. The only sample misclassified by the PCA classification model built on sensor data belongs to patient C13, who performed double exhalation during breath sample acquisition due to physical weakness, which could affect the accuracy of the results (this patient was also misclassified by the PCA model built with the abundances of GC breath biomarkers). The undefined patient U01 (GC patient that responded well to chemotherapy) was plotted in the Controls group. It is important to point out here that the superior results of the sensor test as compared with the model built with the GC biomarkers can be attributed to the fact that the sensor responds to the overall volatiles pattern that includes compounds under the limit of detection or limit of quantification of the GC-MS equipment, yet they can contain important traces of the metabolic changes produced by the GC in the organism.

Similar studies performed on a Chinese population, where the breath samples were measured with an array of 15 chemical gas sensors based on gold nanoparticles functionalized with 11 different organic ligands and bilayers of single-walled carbon nanotubes and organic derivatives, achieved 86% classification accuracy [8].

The significantly superior results obtained in the present study can be attributed to several betterments that we introduced. On one hand, the sensing material was fabricated employing an innovative technique recently reported by the authors, which allows for the fabrication of ultrapure monolayer-capped metal nanoparticles [24]. The main advantage of this technique is that the gold nanoparticles are synthesized in vacuum from the melting of a pure metal (*see* Materials and Methods section), which ensures the fabrication of ultrapure nanoparticles. Sensor's response is thus not cross-influenced by confounding reactions with synthesis residuals, as can be the case of the sensing materials produced by wet-chemistry methods [25], such as those reported in the previous study of gastric cancer performed on a Chinese population [8]. Moreover, we introduced a new sensor that uses a completely new organic functionality (octadecylamine), with high affinity towards long chain aliphatic hydrocarbons (including alkanes and alkenes), due to its hydrocarbon C-C tail. On the other hand, the sensing technique proposed, which comprises 10 sec operation cycles followed by 70 sec stand-by, provides dynamic behavior that allows for fully exploring the reaction kinetics between the sensing material and the breath volatiles under repeated short operation pulses.

An important remark is that, in comparison with other previous studies, our methodology has paramount advantages in terms of both: (i) breath sample collection procedure: the patient breathes normally through the breath sampler instead of performing an extenuating lung wash and providing a deep breath, which can extensively exhaust the patients with gastric affections; (ii) samples measurement: the breath sample is directly injected into the test chamber, without the need to be at first stored in a sorbent tube and t hen heated for releasing the absorbed volatiles, with the inherent loss of VOCs; and (iii) simplicity and reliability of the sensing system: use of only one chemical gas sensor, with simpler operation and data analysis procedures, and less prone to artefacts that can be introduced by a sensors array. Because of these advantages, our test can be performed directly in the hospital where the patient is attended, as it was done in this study.

Whereas the endoscopy is painful, invasive, unpleasant for the patient, not free of risks and not easily available in the developing countries for performing large scale population screening due to its elevated costs and the need of qualified medical staff, the method that we propose in this study uses non-invasive biological samples (*i.e.*, exhaled breath) that are easily collected (the patient just needs to breathe normally through a breath sampler device), it is easy to perform, fast, not expensive, and thus highly suitable for mass-screening of the high risk population. Due to the affordable methodology employed, this test can be easily repeated in case of doubts. Although validation on large cohorts is still absolutely necessary, we can state that our diagnosis test holds excellent potential to be introduced in the future as a large scale prediagnosis method for GC in Colombia, allowing for the selection of the patients who should undergo more detailed analysis (*e.g.*, endoscopy and biopsy). A validation study will be realized as a future step and the results will be published elsewhere.

### Final remark

Although in this study we used a different breath sample collection procedure and different analytical equipment that could produce slightly different results, the studies with patients from China and Latvia that found geographical differences between these two populations were realized by the same research group employing exactly the same breath sampling procedure and the same analytical equipment. Therefore, these differences could be rather attributed to the genetic, lifestyle and alimentation differences among these populations from the World regions with highest GC incidence rates, and could a priori indicate the necessity of developing a personalized breath test adapted to the actual patients living or provenience region.

## MATERIALS AND METHODS

### Patients and breath samples collection

30 volunteers (14 GC, 15 Controls and 1 Undefined) were selected for this study among adult patients attended for gastric complains at Hospital Universitario la Samaritana from the dependencies of Gastrosur S.A. in Bogotá, Colombia. Full information on the patients included in this study is presented in Table 2. The patients were thoroughly explained the aim of this study and the medical procedures to be followed during samples collection, and they were included in the study only after signing the informed consent. The security regulations of the hospital were rigorously applied in the case of both the patients and the medical personnel that took part in the study. Endoscopy tests were performed to each patient in the same day of breath samples collection, before breath sampling, while biopsy tests were further performed whenever necessary. These tests led to the final confirmation of the disease of each patient. In all cases the adenocarcinoma was confirmed by pathological tests.

**Table 2:** Patients information

Patient label	Medical diagnostic	Age	Gender Female (F), Male (M)	Living zone Rural (R), Urban (U)	Medication	Smoker	Histological classification	Location	H. pylori	Study	
										GC-MS	Sensor
**G01**	GC	71	F	R	Tramadol Ranitidine Hyoscine Metoclopramide Omeprazole Dipyrone Hydromorphone	No	Intestinal	Body and Antrum	No	X	X
**G03**	GC	79	F	R	Ranitidine Hyoscine Metoclopramide Omeprazole Captopril Morphine	No	Diffuse	Body and Antrum	No	X	X
**G04**	GC	63	M	R	Omeprazole	No	Intestinal	Antrum	No	X	X
**G06**	GC	66	F	U	No data		Intestinal	Antrum	No	X	-
**G07**	GC	77	M	R	Acetaminophen Tramadol Omeprazole	No	Intestinal	Cardias	No	X	X
**G08**	GC	76	M	R	Sodium Acetaminophen Omeprazole	No	Intestinal	Body and Cardias	No	X	X
**G09**	GC	85	M	U	Omeprazole	No	Intestinal	Body and Cardias	No	X	X
**G10**	GC	79	M	U	Tramadol, Omeprazole Metoclopramide Morphine	No	Intestinal	Cardias	No	X	X
**G11**	GC	66	F	R	Metoclopramide Ranitidine Hyoscine Potassium	No	Intestinal	Antrum	No	X	X
**G12**	GC	61	M	R	Ranitidine metoclopramide Bromide Ipratropium Hyoscine	Yes	Diffuse	Antrum	No	X	X
**G13**	GC	46	M	R	Hydromorphone Hyoscine	Yes	Intestinal	Antrum	No	X	X
**G14**	GC	76	M	U	No data	No	Diffuse	Antrum	No	X	X
**G15**	GC	80	M	R	Furosemide Carvedilol Atorvastatin Ipratropium bromide Spironolactone Enalapril Omeprazole	Yes	Intestinal	Body	No	X	-
**G17**	GC	80	M	U	Omeprazole	Yes	Intestinal	Antrum	No	X	-
**C01**	Gastritis	54	F	R	No data	No	Non-atrophic gastritis	Antrum	Yes	X	X
**C02**	Gastritis	70	M	R	Omeprazole	Yes	Atrophic gastritis	Body and Antrum	No	X	X
**C03**	Gastritis	88	M	R	Naproxen	Yes	Non-atrophic gastritis		No	X	X
**C04**	Gastritis	69	M	U	Tramadol AllopurinolCalcitriol Furosemide Levothyroxine Losartan Omeprazole Propranolol Clonazepam	Yes	Non-atrophic gastritis	Antrum	No	X	X
**C05**	Gastritis	60	F	U	Losartan Omeprazole Hydrochlorothiazide Aluminum hydroxide	No	Non-atrophic gastritis	Antrum	No	X	X
**C06**	Gastritis	56	F	U	Levotiroxin Losartan Metformin	No	Atrophic gastritis	Antrum	Yes	X	X
**C07**	Gastritis/Ulcer	74	F	U	Omeprazole Nifedipine Lovastatin Losartan Levothyroxine Hydrochlorothiazide	No	Non-atrophic gastritis	Antrum	No	X	X
**C08**	Gastritis	62	M	R	Omeprazole Captopril	Yes	Atrophic gastritis	Antrum	Yes	X	X
**C09**	Gastritis	58	F	U	Ranitidine Losartan Hyoscine	No	Atrophic gastritis	Body and Antrum	Yes	X	X
**C10**	Gastritis	77	F	U	Omeprazole aluminum hydroxide	No	Non-atrophic gastritis	Antrum	No	X	X
**C13**	Gastritis	71	F	R	Losartan Omeprazole Loratadine	No	Non-atrophic gastritis	Antrum	Yes	X	X
**C14**	Gastritis	58	F	U	Levothyroxine Atorvastatin OmeprazoleAcetylsalicylic	No	Non-atrophic gastritis	Antrum	Yes	X	X
**C15**	Gastritis	86	M	R	Omeprazole MagnesiumCalcium	No	Atrophic gastritis	Body and Antrum	No	X	X
**C16**	Gastritis	87	M	R	No data	Yes	Atrophic gastritis	Body and Antrum	No	X	X
**C17**	Gastritis	49	M	R	Trimebutine	No	Non-atrophic gastritis	Antrum	No	X	X
**U01**	Satisfactory recovery progress after GC treatment with chemotherapy	75	M	U	No data	No	Atrophic gastritis	Body and Antrum	No	X	X

Histological classification of gastric carcinoma was based on Lauren's criteria.

The patients did not consume any kind of food, drink or tobacco at least for 10 hours before breath samples collection. The samples were acquired employing the BioVOC™ breath sampler device (Markes International, UK). During breath sampling, the patient exhaled normally through a disposable mouthpiece until totally emptying the lungs. The first part of the breath exited through the no-return open end of the breath sampler device, which retained only the last 129 ml part of the breath corresponding to the alveolar air that contains the compounds exchanged by the blood with the lungs and is more probably to contain indices of the gastric lesions. Before any use, each breath sampler was cleaned for 15 min in a solution of 20 ml of disinfectant (Amukina, Spain) dissolved in 1 litter of distilled water, being then left to naturally dry without wiping.

Each patient provided either one or more breath samples, which were measured with the GC-MS system and/or with the chemical gas sensor (*see* Table 2). For the sensing measurements, the breath samples were measured immediately after collection in a hospital room specially accommodated for this purpose. In the case of the GC-MS studies, the breath samples were transferred to storage glass tubes filled with Tenax TA sorbent material suitable for the storage of breath VOCs (ORBO™ 420 Tenax TA sorption tubes purchased from Sigma-Aldrich, Colombia). For increasing the concentration of the VOCs trapped by the sorbent material, two breath samples of the same patient were transferred to the same storage tube. The sorbent tubes were stored at 4°C in a fridge for biological samples before analysis, and shipped to URV (Spain) for performing the GC-MS studies. This analysis was performed six months after samples collection.

### GC-MS analysis and biomarkers identification

For the identification of the breath biomarkers associated with gastric cancer, the samples were analyzed with a Quadrupole Time-of-Flight Gas Chromatography/Mass Spectrometry (GC/Q-TOF) analytical instrument (Agilent G7200AA), which represents the state-of-the-art in GC-MS analysis. For performing the GC-MS measurements, at first the Tenax material from the sorbent tube was transferred into a 20 mL glass vial that was sealed and heated at 100°C on a hotplate for desorbing the trapped breath volatiles. The Solid Phase Micro-Extraction (SPME) technique was used to preconcentrate the volatiles released by the sorbent material in the headspace formed above the Tenax material. The volatiles captured by the SPME DVB/Carboxen/PDMS fiber were injected into the GC/Q-TOF port for analysis, using 26.25 min runtime.

The GC/Q-TOF system was operated in the splitless mode, and the following extraction and chromatographic conditions were used: Extraction time: 20 min; Extraction temperature: 100°C; Desorption time: 2 min; Desorption temperature: 250°C. The oven temperature profile used was: a) 10 min at 50 °C; b) Ramp of 10°C/min until 155°C; c) Ramp of 20°C/min until 270°C; d) 10 min at 250°C.

After realizing the deconvolution of the chromatograms acquired employing the Unknown Analysis software programed in the automatic mode, with the match factor value set to 80, in each breath sample were identified up to 650 compounds that were determined employing the NIST 14 mass spectral library. The most abundant VOCs are presented in Supplementary Table 2 from the Supplemental Material. The putative breath biomarkers for GC were found applying the statistical t-test using the standard cut-off value α = 0.05, corresponding to 95% confidence interval. The similarity index showing the accuracy of compounds identification and alternative compounds identifications proposed by the software are presented in Supplementary Table 3 from the Supplemental Material. Patients sex (male/female), age (below/over 60 years old), smoking habit and the presence/absence of *helicobacter pylori* infection were considered as confounding factors for disregarding from the initial list of putative biomarkers those that could be affected by factors not related with the disease itself.

### Chemical gas sensor and sensing measurements

**Figure 4:**
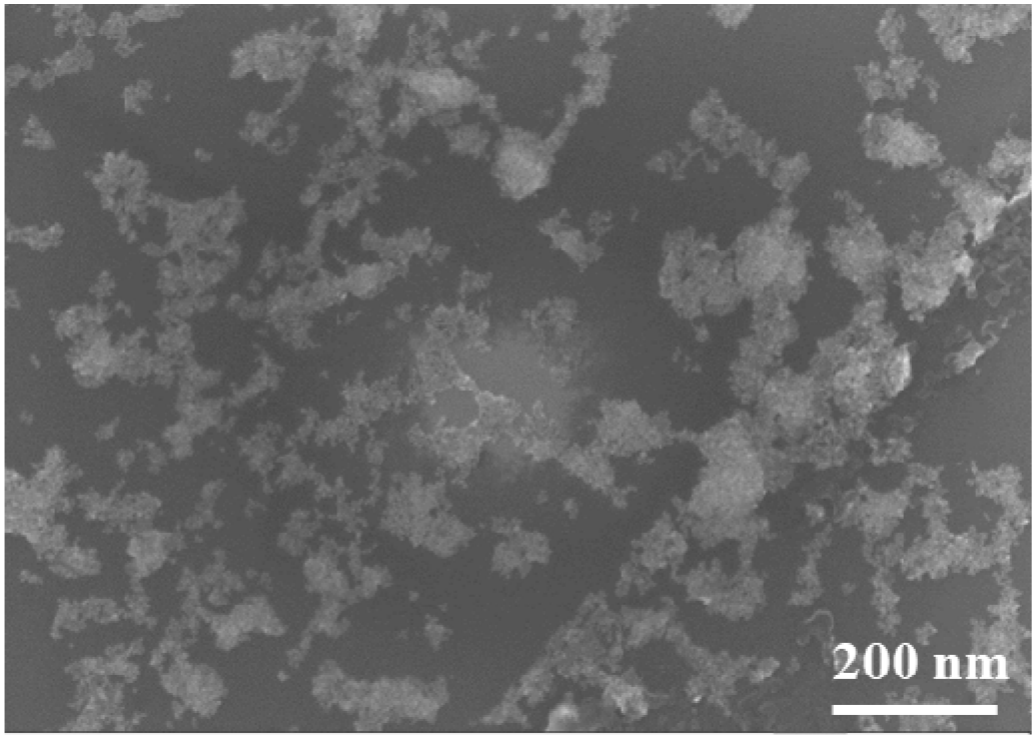
Surface electron microscopy (SEM) image of the AuNP-octadecylamine sensing film This image was acquired with a Zeiss LEO 1550 High Resolution Scanning Electron Microscope (HR-SEM), using a field emission gun as electron source, an acceleration voltage of 10 kV, and 100,000 magnification value.

The sensing device was fabricated on a silicon substrate, where two parallel gold electrodes were patterned by standard chemical etching. The sensing material was synthesized by depositing at first a monolayer of ultrapure monodispersed gold nanoparticles in the 15 μm gap between the tips of the gold electrodes, employing the advanced gas deposition (AGD) system (Ultra Fine Particle Equipment, ULVAC Ltd, Japan [26]). For this, a high purity gold piece (99.999% purity) was placed inside an induction coil in the evaporation chamber of the AGD equipment and heated over the melting point of gold in an inert He gas atmosphere. Au atoms released from the surface of the molten gold metal piece formed gold nanoparticles by collision, which were carried upwards through a narrow transfer pipe (3 mm diameter) by the He carrier gas introduced underneath, to the deposition chamber of the AGD equipment where the sensing substrate was placed on a movable support. Because of the pressure difference between the two AGD chambers (90.8 mbar in the evaporation chamber and 0.09 mbar in the deposition chamber, respectively), the AuNP impinged at high speed on the sensor surface, where they got strongly adhered. The experimental conditions (pressures in the two AGD chambers and speed of the movable support set to 1 mm/sec) were adjusted such that to obtain the deposition of dispersed AuNP. In the next step, the sensor was introduced for 1 hour in a solution of 100 μL of octadecylamine dissolved in 20 ml ethanol and then heated for 1 hour at 50°C in ambient atmosphere in a conventional oven for ethanol solvent evaporation, using a temperature ramp of 10°C/min. This process produced the functionalization of the AuNP with octadecylamine, forming a network-like structure (Figure 4) where the AuNP ensures the electrical conduction through the sensing film, while the organic functionalities provide reaction sites for the volatiles to which the sensor is exposed.

For performing the sensing measurements, the sensor was placed inside a Teflon test chamber (26 cm^3^ inner volume) provided with two orifices for sample inlet and sample outlet, respectively. The breath sample was directly injected from the BioVOC into the sensor test chamber by slowly pushing the plunger during 10 sec. Each sensing measurement comprised the following cycles: (a) 5 min stabilization under steady state conditions; (b) 10 min exposure to the breath sample under steady state conditions; (c) 5 min for cleaning the sensor surface and purging the test chamber using continuous synthetic dry air (1 l/min flow rate). During the measurements, the sensor was operated at 5V in a sequential mode (6 cycles of 10 sec ON followed by 70 sec OFF) in order to fully exploit the absorption/desorption reaction kinetics between the sensing material and the breath volatiles. A high precision power source (B2902A, Keysight Technologies, Hungary) was used for applying the operating voltage, and a high resolution data acquisition system (34972A LXI/Data Acquisition, Keysight Technologies, Hungary) for acquiring the current through the sensor for further analysis.

### Data analysis

**Figure 5:**
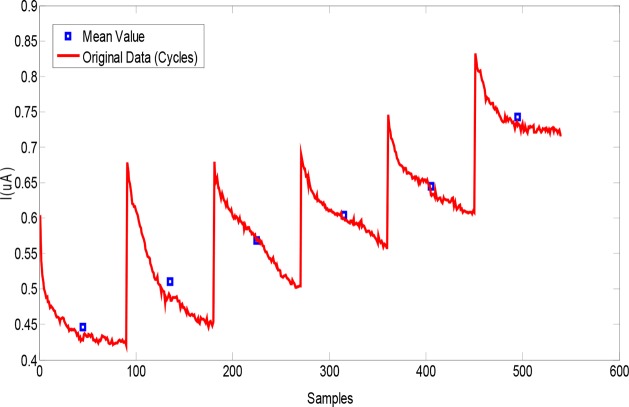
Typical sensor response to an exhaled breath sample (red curve) and the features extracted (blue points)

Data reduction was performed at first for reducing the size of the information acquired by the sensor from the 540 initial data points to 6 data points (1 point per cycle), corresponding to the mean value of all the current values acquired in that cycle (*see* Figure 5). These features were next used as input data to the pattern recognition algorithm.

Principal Component Analysis (PCA) was used for building the samples classification models. PCA is a multivariate data analysis method that is widely useful for data classification [27, 28]. This powerful data analysis tool was selected in this application since it is an effective linear unsupervised method able to extract the most relevant information from the dataset and project that information into a low-dimensional plane using the scores plot. In this study, the dataset was auto-scaled before applying the PCA in order to make all the measurements equally weighted.

The prediction accuracy of the PCA model was calculated as the number of correctly grouped samples over the total number of samples employed in the analysis. The sensitivity was calculated as number of true positives over true positives plus false negatives, and the specificity as the number of true negatives over true negatives plus false positives.

## SUPPLEMENTARY MATERIALS FIGURES AND TABLES





## Abbreviations

GC: Gastric Cancer
VOCs: Volatile Organic Compounds
GC-MS: Gas-Chromatography/Mass-Spectrometry
GC/Q-TOF: Quadrupole Time-of-Flight Gas Chromatography/Mass Spectrometry
SPME: Solid Phase Micro-Extraction
PCA: Principal Component Analysis
AuNP: Gold Nanoparticles
H. pylori: Helicobacter pylori
IARC: International Agency for the Research of Cancer
EPA: Environment Protection Agency
POCP: Photochemical Ozone Creation Potential
URV: Rovira i Virgili University
