# Supplementary material for: Exhaled breath analysis for gastric cancer diagnosis in Colombian patients
Source: Oncotarget. 2018 Jun 22;9(48):28805–17. doi: 10.18632/oncotarget.25331 (PMC6034740; doi:10.18632/oncotarget.25331)
Supplement: Supplementary file 1 [file oncotarget-09-28805-s001.pdf]

Exhaled breath analysis for gastric cancer diagnosis in Colombian patients

SUPPLEMENTARY MATERIALS

PCA analysis performed with the breath biomarkers

The variance captured by each PC for the PCA analysis performed with the breath biomarkers for gastric cancer identified in this study is presented in Supplementary Table 1.

Exhaled breath analysis

The GC-MS studies identified up to 650 volatile organic compounds in the breath of each volunteer. The most abundant of them are presented in Supplementary Table 2.

The similarity index of the putative biomarkers identified from the statistical analysis of the breath samples is provided in Supplementary Table 3, as well as alternative compounds identifications for each biomarker proposed by the software employed to analyse the chromatographic data.

Biomarkers abundances in the breath sample of every volunteer

Patients classification based on confounding factors achieved by the PCA model built with sensor's responses

Supplementary Figure 7 shows patients classification based on the most important confounding factors that can be found in the breath of patients with gastric diseases provided by the PCA model built with sensor's responses, confirming that these confounding factors do not affect patients classification based on gastric cancer sickness.

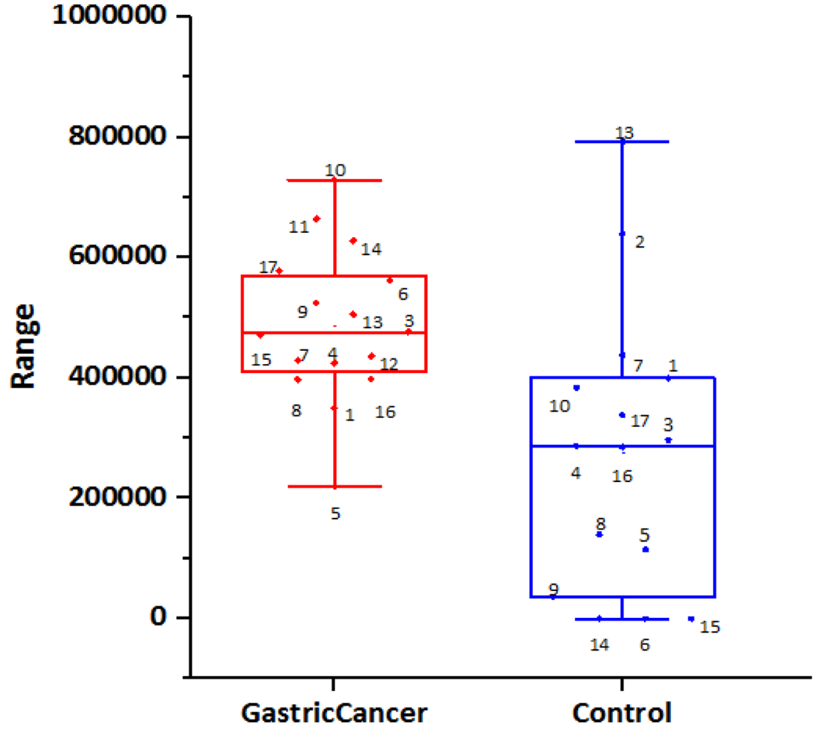

Supplementary Figure 1: Boxplot diagram for *trans-2,2-dimethyl-3-decene*.

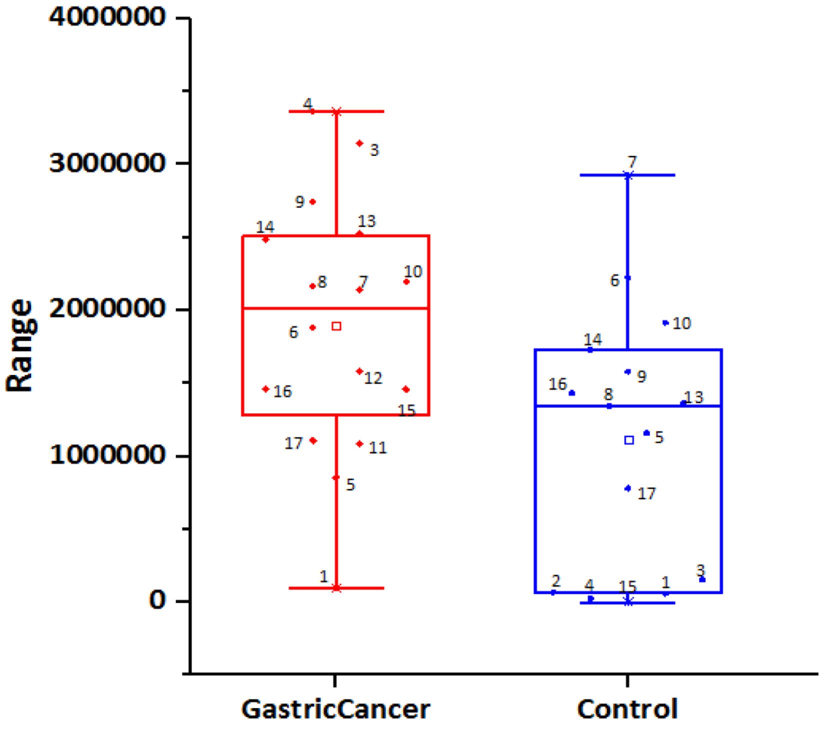

Supplementary Figure 2: Boxplot diagram for *octadecane*.

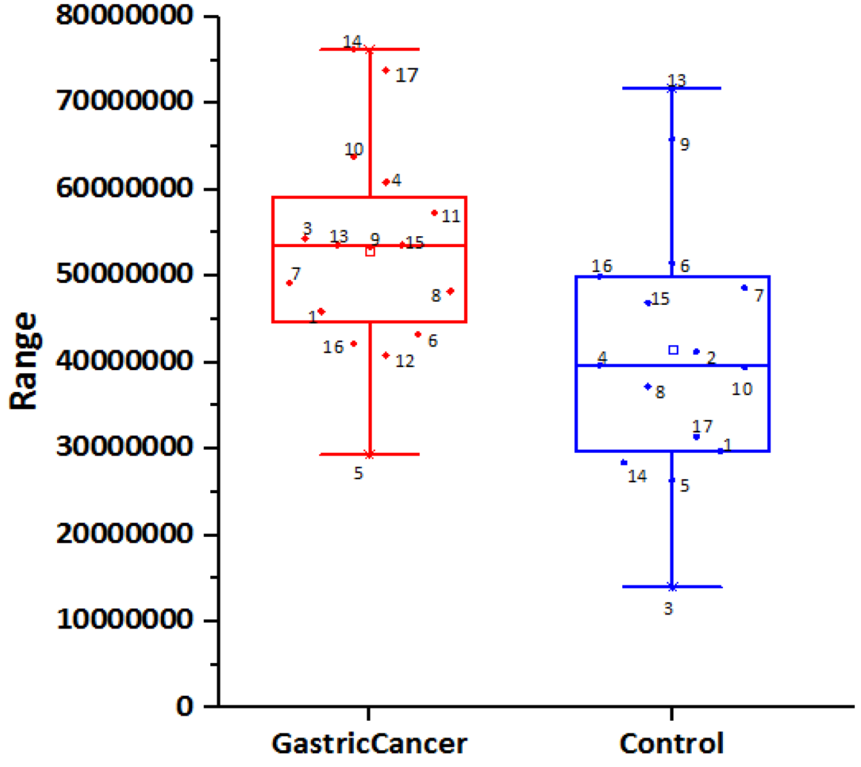

Supplementary Figure 3: Boxplot diagram for *m-xylene*.

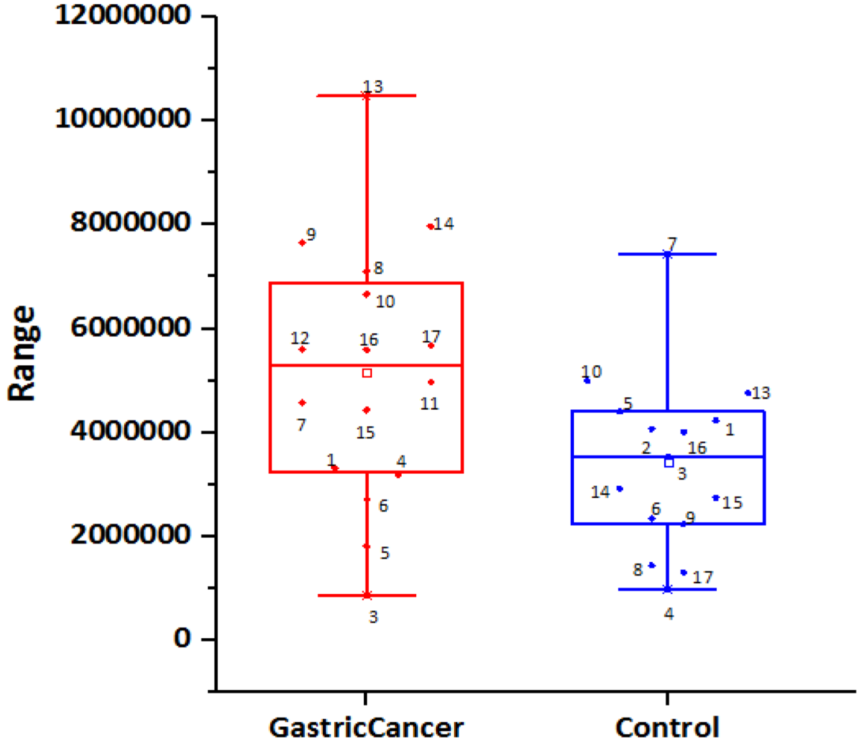

Supplementary Figure 4: Boxplot diagram for *hexadecane*.

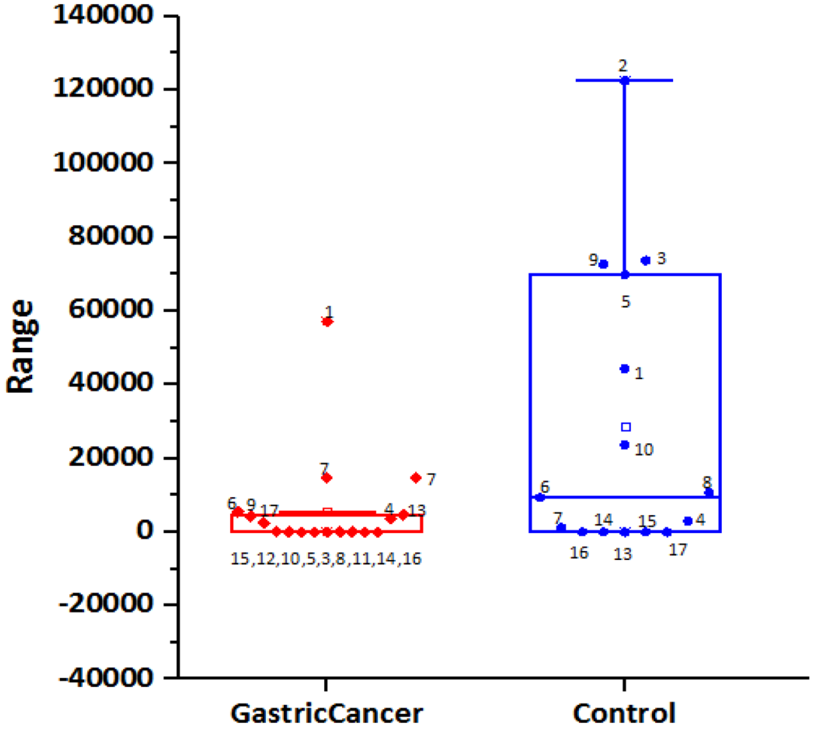

Supplementary Figure 5: Boxplot diagram for *1-cyclohexyl-2-(cyclohexylmethyl) pentane*.

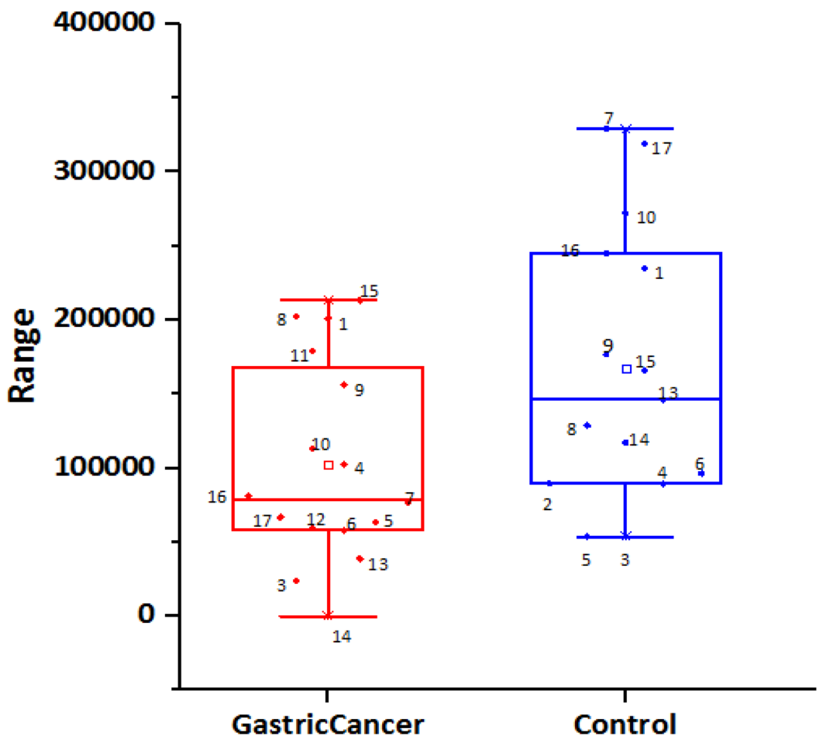

Supplementary Figure 6: Boxplot diagram for *eicosane*.

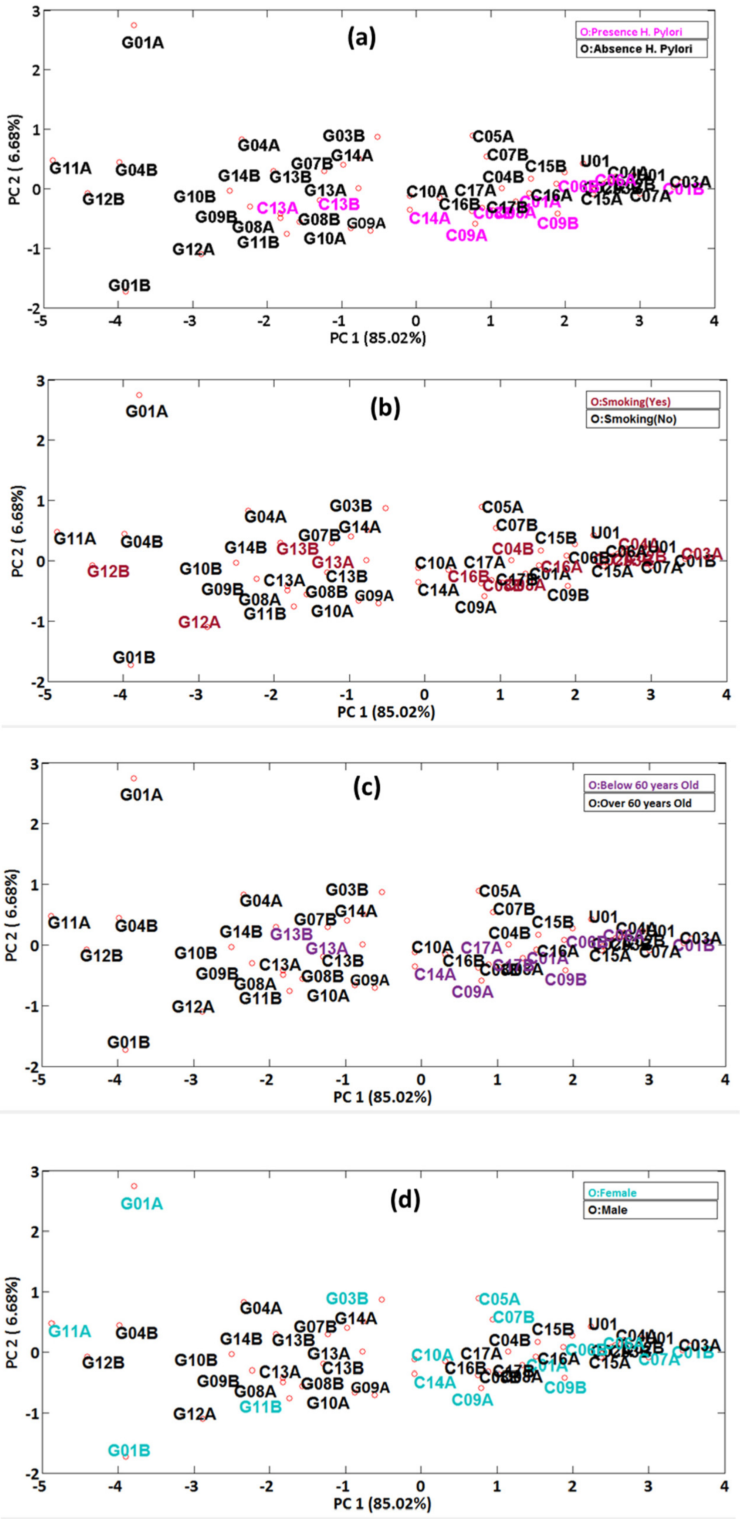

Supplementary Figure 7: PCA scores plot obtained from sensor's responses when the following classifications were assessed: **(a)** Presence/absence of *H. pylori* infection; **(b)** Smoking habit; **(c)** Age; **(d)** Gender.

Supplementary Table 1: Variance captured by each PCA in the PCA model performed with the breath biomarkers

Principal Component	%Variance (This PC)	%Variance (Cumulative)
1	40.61	40.61
2	18.57	59.19
3	16.54	75.72
4	10.44	86.16
5	8.19	94.36
6	5.64	100

Supplementary Table 2: Most abundant volatile organic compounds in volunteers breath

See Supplementary File 1

Supplementary Table 3: Information about the putative biomarkers for gastric cancer identified in this study, and alternative compounds proposed by the software

No.	Compound	Mass to charge ratio (m/z)	Retention time	Match factor (%)	Alternative compounds
1	Trans-2,2-dimethyl-3-decene	69.0716	7.6237	68.9	Undec-10-ynoic acid, dodecyl ester
					2-Undecanethiol, 2-methyl-
					4-Nonene, 5-butyl-
					Cyclododecane
2	Octadecane	71.0873	10.8352	80.8	Phytol
					(E)-Hexadec-2-enal
					1-Decanol, 2-hexyl-
3	M-xylene	91.0576	2.8311	94.6	P-xylene
					O-xylene
					Benzene, 1,3-dimethyl-
4	Hexadecane	43.0559	8.1518	76.6	1-Octanol, 2-butyl-
					1-Decanol, 2-ethyl-
					Decyl octyl ether
					2-Isopropyl-5-methyl-1-heptanol
					1-Hexanol, 5-methyl-2-(1-methylethyl)-
					Tridecane, 6-methyl-
5	1-Cyclohexyl-2-(cyclohexylmethyl) pentane	153.1655	5.3618	71.4	Cyclohexane, 2,4-diisopropyl-1,1-dimethyl-
					Cyclohexane, 1,5-diisopropyl-2,3-dimethyl-
6	Eicosane	215.1779	13.2262	77.3	Formic acid, 3,7,11-trimethyl-1,6,10-dodecatrien-3-yl ester
					1-Heptatriacotanol
					Limonen-6-ol, pivalate
					cis-Z-.alpha.-Bisabolene epoxide
