# Supplementary material for: Exhaled breath analysis for gastric cancer diagnosis in Colombian patients
Source: Oncotarget. 2018 Jun 22;9(48):28805–17. doi: 10.18632/oncotarget.25331 (PMC6034740; doi:10.18632/oncotarget.25331)
Supplement: Supplementary file 2 [file oncotarget-09-28805-s002.pdf]

**Supplementary Table 2.** Most abundant volatile organic compounds in volunteers breath

| No. | Compound | Mass to charge ratio (m/z) | Retention time |
|---|---|---|---|
| 1 | Toluene | 92.0650 | 1.9343 |
| 2 | Benzene, 1,3-dimethyl- | 91.0576 | 2.8396 |
| 3 | Styrene | 78.0491 | 3.0782 |
| 4 | Nonane, 4,5-dimethyl- | 57.0723 | 5.2319 |
| 5 | Hexadecane | 85.1037 | 8.3898 |
| 6 | Trichloromethane | 85.1037 | 1.2379 |
| 7 | 3-Eicosene, (E)- | 69.0721 | 8.7487 |
| 8 | 3-Hexadecene, (Z)- | 69.0721 | 8.8650 |
| 9 | Cyclopentasiloxane, decamethyl- | 355.0818 | 6.6081 |
| 10 | 1-Octanol, 2-butyl- | 57.0723 | 8.9749 |
| 11 | Cyclopentane, 1,1,3,4-tetramethyl-, cis- | 69.0721 | 5.5452 |
| 12 | Cyclooctane, 1,4-dimethyl-, cis- | 69.0721 | 5.6049 |
| 13 | Decane, 2,4-dimethyl- | 57.0723 | 5.3111 |
| 14 | Dodecane, 4,6-dimethyl- | 57.0723 | 7.8907 |
| 15 | Nonane, 2,6-dimethyl- | 57.0723 | 4.6062 |
| 16 | Benzyl alcohol | 108.0601 | 4.9184 |
| 17 | Silane, cyclohexyldimethoxymethyl- | 75.0276 | 6.7268 |
| 18 | Nonane, 2,2,4,4,6,8,8-heptamethyl- | 57.0723 | 9.0469 |
| 19 | Undecane, 4,7-dimethyl- | 57.0723 | 5.9463 |
| 20 | 1-Hexanol, 5-methyl-2-(1-methylethyl)- | 57.0723 | 8.5037 |
| 21 | (S)-(+)-5-Methyl-1-heptanol | 57.0723 | 4.8112 |
| 22 | Carbonic acid, eicosyl vinyl ester | 57.0723 | 9.6588 |
| 23 | 3-Octadecene, (E)- | 69.0721 | 10.2267 |
| 24 | D-Limonene | 93.0717 | 4.8523 |
| 25 | Cyclohexasiloxane, dodecamethyl- | 342.0264 | 9.0247 |
| 26 | 2-Propenoic acid, butyl ester | 73.0313 | 3.0946 |
| 27 | Decyl octyl ether | 85.1037 | 9.1359 |
| 28 | Hexanal, 2,2-dimethyl- | 72.0904 | 1.1785 |
| 29 | Sulfurous acid, pentyl tridecyl ester | 57.0723 | 10.6132 |
| 30 | Benzene, (1-methylethyl)- | 120.0971 | 3.9394 |
| 31 | Tridecane, 6-methyl- | 57.0723 | 9.8464 |
| 32 | Carbonic acid, octyl vinyl ester | 43.0561 | 5.2645 |
| 33 | Pentanoic acid | 60.0226 | 4.2760 |
| 34 | Performic acid, trimethylsilyl derivative | 119.0884 | 7.4125 |
| 35 | Ethylbenzene | 91.0576 | 2.7586 |
| 36 | 1-Heptanol, 2,4-diethyl- | 69.0721 | 10.9604 |
| 37 | Decane, 2,3,5,8-tetramethyl- | 85.1037 | 10.6757 |
| 38 | Propanoic acid, butyl ester | 75.0276 | 3.2334 |
| 39 | Octane, 4-methyl- | 56.0635 | 2.7445 |
| 40 | Tetradecane, 1-iodo- | 85.1037 | 11.1645 |
| 41 | Hexane, 3,3-dimethyl- | 57.0723 | 6.0652 |
| 42 | 1-Undecene, 4-methyl- | 85.1037 | 5.7675 |
| 43 | Butoxyacetic acid | 57.0723 | 2.9497 |
| 44 | Acetic acid, pentyl ester | 61.0126 | 1.2174 |
| 45 | .alpha.-Methylstyrene | 117.0725 | 4.2128 |
| 46 | Decane, 3,3,5-trimethyl- | 57.0723 | 7.9914 |
| 47 | Decane, 2,5,6-trimethyl- | 57.0723 | 4.4157 |
| 48 | Dodecane, 5-methyl- | 126.1419 | 9.7921 |

| | | | |
|---|---|---|---|
| 49 | Sulfurous acid, dodecyl hexyl ester | 85.1037 | 9.2894 |
| 50 | Cycloheptasiloxane, tetradecamethyl- | 281.0557 | 11.1872 |
| 51 | Phytol | 57.0723 | 6.9176 |
| 52 | 1-Octen-3-ol | 72.0904 | 2.1513 |
| 53 | 2-Isopropyl-5-methyl-1-heptanol | 84.0951 | 10.7524 |
| 54 | Carbonic acid, nonyl vinyl ester | 57.0723 | 6.7449 |
| 55 | Cyclotrisiloxane, hexamethyl- | 207.0358 | 2.3140 |
| 56 | Octane, 2,4,6-trimethyl- | 57.0723 | 4.1015 |
| 57 | Cyclohexane, 1,2-dimethyl-3-pentyl-4-propyl- | 153.1666 | 9.2310 |
| 58 | 1-Undecanol | 161.1354 | 10.8531 |
| 59 | 1,11-Dodecadiene | 67.0555 | 5.8987 |
| 60 | 1-Decanol, 2-ethyl- | 57.0723 | 6.5501 |
| 61 | Heptane, 2,3,6-trimethyl- | 57.0723 | 3.9042 |
| 62 | Dodecane, 1-iodo- | 85.1037 | 10.8425 |
| 63 | 1,7-Octadiene, 2,7-dimethyl- | 56.0635 | 4.4620 |
| 64 | Pentane, 2,2,3,4-tetramethyl- | 57.0723 | 3.1385 |
| 65 | Sulfurous acid, dodecyl pentyl ester | 85.1037 | 8.9351 |
| 66 | Oxalic acid, 6-ethyloct-3-yl isohexyl ester | 85.1037 | 9.9978 |
| 67 | Heptacosane | 57.0723 | 11.6067 |
| 68 | Cyclohexanol, 2-(1,1-dimethylethyl)- | 82.0787 | 8.6192 |
| 69 | 2-Octene, 4-ethyl-, (E)- | 69.0721 | 9.0865 |
| 70 | Sulfurous acid, isohexyl 2-pentyl ester | 85.1037 | 6.9426 |
| 71 | Octane, 6-ethyl-2-methyl- | 119.0884 | 5.1329 |
| 72 | 3,4-Dihydroxyphenylglycol, 4TMS derivative | 355.0818 | 12.6697 |
| 73 | 2,3-Dimethyl-3-heptene, (Z)- | 56.0635 | 11.1530 |
| 74 | Hexane, 2,3,5-trimethyl- | 85.1037 | 2.3394 |
| 75 | Decane, 2,3,8-trimethyl- | 85.1037 | 8.8201 |
| 76 | Propanoic acid, 2,2-dimethyl-, pentyl ester | 59.0504 | 4.4553 |
| 77 | Benzene, propyl- | 91.0576 | 3.8414 |
| 78 | Acetic acid, TMS derivative | 75.0276 | 8.3543 |
| 79 | 2-Piperidinone, N-[4-bromo-n-butyl]- | 85.1037 | 9.9231 |
| 80 | Cyclopropane, 1-ethyl-2-heptyl- | 56.0635 | 4.9033 |

The similarity index of the putative biomarkers identified from the statistical analysis of the breath samples is provided in Table S3, as well as alternative compounds identifications for each biomarker proposed by the software employed to analyse the chromatographic data.
